# RAD51 wrestles with SUMO

**DOI:** 10.1080/23723556.2022.2054263

**Published:** 2022-03-31

**Authors:** Younghoon Kee, Jung-Hee Lee, Ho Jin You

**Affiliations:** a Department of New Biology, Daegu Gyeongbuk Institute of Science and Technology (DGIST), Daegu, Republic of Korea; b Department of Cellular and Molecular Medicine, Chosun University School of medicine, Gwangju, Republic of Korea

**Keywords:** RAD51, SUMO

## Abstract

RAD51 loading onto chromatin is a key step during the homologous recombination (HR) repair. We recently reported a new mode of RAD51 regulation, which is mediated by TOPORS E3 SUMO ligase and RAD51 SUMOylation. ATM/ATR-induced phosphorylation of TOPORS is necessary for this event, revealing a new role of these master DNA damage response kinases in HR repair.

DNA double-strand breaks (DSBs) are dangerous lesions that can result in large-scale chromosomal translocation, loss of genomic information, and cancer. It is no surprise that DSB repair requires sophisticated regulations, in particular, during the homologous recombination (HR) repair, to ensure that lost genetic information is restored with high accuracy.

During the early events of HR repair, single stranded DNA (ssDNA) overhangs are generated at DSB ends by concerted actions of nucleases. The ssDNA overhangs are immediately coated by Replication Protein A (RPA) proteins, which are then replaced by RAD51 recombinase to form a nucleoprotein filament competent for invasion of the homologous DNA template. The replacement by RAD51 to form RAD51-ssDNA filament (we refer this step as ‘RAD51 loading’) is a rate-limiting step that is assisted by many co-factor proteins; in vertebrates, notable factors include BRCA1, PALB2, and BRCA2, whose sequential interplay eventually brings in RAD51 to the DSB ends. Some of the RAD51 loading factors are additionally modified by the master DNA damage response kinases ATM and ATR; ATM phosphorylates BRCA1,^1^ while ATR-mediated phosphorylation of PALB2 induces the BRCA1-PALB2 interaction^2^ and PALB2-RAD51 interaction.^3^ These findings suggest that RAD51 interaction with the co-factors is inducibly regulated, rather than being constitutively bound. Although these studies unequivocally find that ATR inhibition reduces the RAD51 loading and HR repair, it is likely that full repertoire of HR-promoting functions of ATR is yet to be uncovered.

Protein modification by SUMO (Small Ubiquitin-like Modifier) has emerged as an important regulatory mechanism for altering signaling or protein functions. Unlike ubiquitination, it does not directly lead to protein destruction but rather it enhances protein–protein interactions. Requirement for SUMOylation in HR repair has been documented; for instance, SUMO proteins and the E2 SUMO-conjugating enzyme UBC9 localize at DSB sites.^4^ A SIM (*S*UMO-*I*nteraction *M*otif) within RAD51 regulates HR,^5^ but whether the SUMO conjugation system directly targets RAD51 for modification has been unknown.

We recently reported that RAD51 is SUMOylated at K57 and K70 residues.^6^ The SUMOylation is mediated by TOPORS (topoisomerase 1-binding arginine/serine-rich protein) SUMO E3 ligase, UBC9, and SUMO1 (but not SUMO2 or SUMO3). SUMO1 is not known to form poly-SUMO chains, and our data suggest that two SUMO1 moieties are conjugated at two different residues. The interaction between TOPORS and RAD51 can be detected constitutively (it was found via yeast two hybrid screen), but the interaction is enhanced by phosphorylation by either ATM or ATR; using mass spectrometry, we identified that TOPORS is phosphorylated upon IR at Thr515 residue, an ATM/ATR target site (S/TQ). The phosphorylation at this residue is reduced by inhibiting either ATM or ATR. Importantly, expressing the phosphorylation-defective mutant of TOPORS (T515A) led to a decreased association with RAD51, and consequently, decreased SUMOylation of RAD51. These highlight that the IR-induced RAD51 SUMOylation is regulated by the phosphorylation of TOPORS. Consistently, expressing the phosphorylation-deficient TOPORS or SUMOylation-deficient RAD51 mutant failed to support the normal HR repair capacity, compared to cells expressing wild-type counterparts. Importantly, the ability of SUMOylation-deficient RAD51 mutant in association with its crucial recruiter BRCA2 is reduced, explaining the HR deficiency. These results reveal that RAD51 loading can be regulated by ATM/ATR kinases independently of previously known regulations such as phosphorylating RAD51-loading factors or via their effector kinases CHK1/CHK2 that regulate the RAD51 activity^7^ (Figure 1).

**Figure 1.**
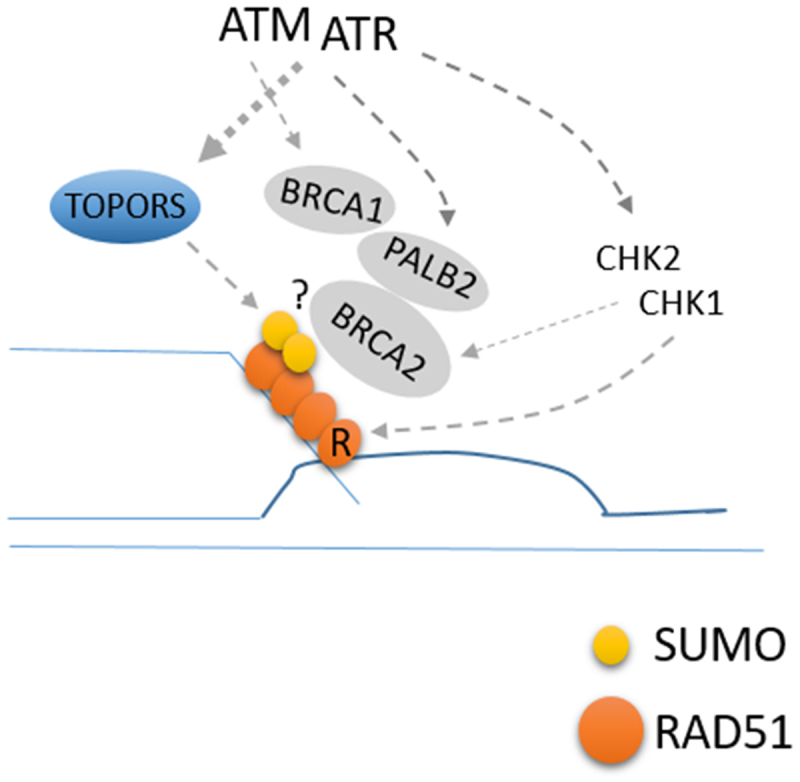
Roles of ATM/ATR kinases in promoting RAD51 chromatin loading. ATM/ATR induces chromatin loading of RAD51 and HR repair, in part, by TOPORS phosphorylation and RAD51 SUMOylation.

Several questions remain to be answered. What is the exact role of the SUMOylation? Although purified RAD51 and BRCA2 directly interact, SUMO may enhance the interaction; it is possible that BRCA2 contains a cryptic SIM, in close proximity to the RAD51-binding BRC repeats, in a way that the SIM-SUMO interaction may facilitate the handover of RAD51 to the BRC repeat regions of BRCA2. Alternatively, the SUMOlyation may reduce RAD51’s ability to bind to random DNA, in order to correctly target RAD51 to the ssDNA overhangs, or inhibit the oligomerization between individual RAD51 monomers^8^ for them to bind to BRCA2. It is also possible that the SUMO moiety regulates other factors that assist RAD51 loading, such as PALB2. Further studies are needed to obtain the mechanistic insight.

Does SUMOylation of RAD51 regulate the dissociation of RAD51-ssDNA filament? As much as RAD51 loading is crucial in HR, so is its timely removal from chromatin. The removal may be regulated by SENP2 de-SUMOylating enzyme, which we found to remove the SUMO moieties from RAD51. Our experimental setup involved knockdown or overexpression of SENP2, thus it is not yet clear what endogenous signal induces the SENP2-mediated de-SUMOylation of RAD51. It is, however, reasonable to think that there is an active signal to induce the de-SUMOylation and its removal from chromatin. In this regard, RAD51 ubiquitination by RFWD3 E3 ligase has been suggested as one mechanism to remove RAD51 from chromatin in late-HR step.^9^ It is interesting to speculate that the de-SUMOylation of RAD51 by SENP2 functionally wires the RAD51 ubiquitination to induce the timely removal of RAD51. We also speculate that de-SUMOylation of RAD51 precedes the access by RAD54, which stimulates the disassembly of RAD51 filament.

Lastly, does TOPORS mutation cause *Fanconi Anemia* (FA)-like phenotype? *RAD51* gene itself (designated as *FANCR*) or many genes encoding the regulators of RAD51, such as *BRCA1*, *PALB2, BRCA2, RAD51C, XRCC2*, and *RFWD3*, are all mutated in FA individuals.^10^ In theory, TOPORS may fall into the category, given the importance of SUMOylation in RAD51 function. Loss of function mutation in TOPORS is likely to be embryonically lethal due to other effects, but there may be hypomorphic variants of TOPORS that mildly disrupt the HR repair and manifest the FA-like phenotypes in human. At least in cellular model, TOPORS knockdown causes sensitivity to DNA interstrand crosslinker Mitomycin C, a classical phenotype of FA.

In all, our work revealed a new regulation for RAD51-dependent HR repair and highlight the emerging roles of SUMO in DNA repair.
